# Cardiovascular function in women with previous gestational diabetes mellitus and dysglycemia at 5 months postpartum

**DOI:** 10.1002/uog.29285

**Published:** 2025-06-30

**Authors:** T. Mansukhani, S. Lausegger, G. Eisenkolb, S. Eniste, C. Gomez Fernandez, K. H. Nicolaides, M. Charakida

**Affiliations:** ^1^ Harris Birthright Research Centre for Fetal Medicine, King's College Hospital London UK; ^2^ Faculty of Medicine Complutense University of Madrid Madrid Spain; ^3^ Department of Women and Children's Health School of Life Course and Population Sciences, King's College London London UK; ^4^ School of Biomedical Engineering and Imaging Sciences, King's College London UK; ^5^ 2nd Department of Pediatrics National and Kapodistrian University of Athens Athens Greece

**Keywords:** diabetes, dysglycemia, echocardiography, gestational diabetes, ophthalmic artery Doppler, prediabetes, pulse‐wave velocity

## Abstract

**Objective:**

To examine the cardiovascular profile of women at 5 months postpartum who developed gestational diabetes mellitus (GDM), and to determine whether this profile differs between those with dysglycemia and those who are normoglycemic.

**Methods:**

A postnatal follow‐up clinic was attended by 770 women who developed GDM. At this visit, we recorded maternal demographic characteristics, obstetric and medical history, details regarding pregnancy outcome, weight, height, upper arm and waist circumference, lipid profile and hemoglobin A1c. We also performed a 75‐g oral glucose tolerance test and extensive cardiovascular assessment, including Doppler measurement of the ophthalmic arteries, carotid‐to‐femoral pulse‐wave velocity and echocardiography. Multivariable logistic regression was performed to assess which factors were associated with dysglycemia. Calibration of the model was assessed using the Hosmer–Lemeshow test and its discrimination was evaluated using receiver‐operating‐characteristics (ROC) curve analysis.

**Results:**

At a median of 5‐months postpartum, more than half of the 770 women had dysglycemia, including 368 (47.8%) with prediabetes and 34 (4.4%) with Type‐2 diabetes mellitus. In the multivariable analysis, the risk factors for dysglycemia were Black ethnicity (adjusted odds ratio (aOR), 2.61 (95% CI, 1.71–3.98)), South Asian ethnicity (aOR, 1.81 (95% CI, 1.18–2.78)), East Asian ethnicity (aOR, 2.46 (95% CI, 1.35–4.48)), mixed ethnicity (aOR, 2.68 (95% CI, 1.19–6.04)), diagnosis of GDM < 24 weeks' gestation (aOR, 2.15 (95% CI, 1.34–3.45)), treatment of GDM with insulin (aOR, 1.96; 95% CI, 1.26–3.05), postnatal body mass index (BMI) (aOR, 1.47 (95% CI, 1.05–2.06)), central diastolic blood pressure (aOR, 1.42 (95% CI, 1.01–2.00)), mitral peak early diastolic flow velocity to early mitral annulus diastolic velocity ratio (E/e′) (aOR, 1.31 (95% CI, 1.00–1.80)) and total peripheral resistance (aOR, 0.61 (95% CI, 0.44–0.86)). The calibration of the model was *P* = 0.397 and the discrimination was indicated by an area under the ROC curve of 0.707 (95% CI, 0.670–0.743).

**Conclusion:**

These findings suggest that women who developed GDM and had persistent dysglycemia at 5 months postpartum have a distinct phenotype which is characterized by increased BMI and signs of cardiovascular strain, including elevated central blood pressure and reduced left ventricular relaxation. These findings suggest that, among women who developed GDM, those with persistent dysglycemia appear to be at increased cardiovascular risk. Further studies are needed to assess whether targeting weight loss with lifestyle or pharmacological interventions can reduce the risk not only for Type‐2 diabetes mellitus, but also for later cardiovascular disease. © 2025 The Author(s). *Ultrasound in Obstetrics & Gynecology* published by John Wiley & Sons Ltd on behalf of International Society of Ultrasound in Obstetrics and Gynecology.

## INTRODUCTION

Epidemiological studies have reported that, although gestational diabetes mellitus (GDM) is a transient condition, its development is associated with a significantly increased risk of Type‐2 diabetes mellitus (DM). Cumulative incidence rates of subsequent Type‐2 DM vary widely, ranging from 2.6% to > 70%, from as early as 6 weeks to as late as 28 years postpartum^1^, ^2^. The risk peaks within the first 5 years after delivery and stabilizes after 10 years postdelivery^2^, ^3^.

Beyond development of diabetes, GDM is also associated with an increased risk of cardiovascular disease later in life^4^, ^5^. A pooled analysis of over 5.3 million women showed a 2.3‐fold increase in cardiovascular events within the first decade postpartum in those who had GDM, and a large cohort study reported a 40% higher risk of cardiovascular disease, particularly in women with prepregnancy obesity or a maternal history of cardiovascular disease^4^, ^6^. A recent meta‐analysis indicated that the link between GDM and major cardiovascular events is independent of the development of Type‐2 DM, suggesting distinct underlying mechanisms^4^. These findings emphasize the need for regular postpartum screening and better characterization of risk phenotypes to understand the pathways leading to Type‐2 DM and cardiovascular disease in women with a history of GDM.

To address this need, we have established a routine 5‐month follow‐up clinic in our center for women who developed GDM, with the aim to monitor their cardiometabolic health and postnatal outcomes longitudinally. In a recent study of 558 women who attended this clinic, we found that a high proportion had dysglycemia, defined as prediabetes or Type‐2 DM. Multivariable logistic regression analysis showed that significant predictors of dysglycemia were Black ethnicity, a severe form of GDM, with diagnosis < 24 weeks' gestation, and treatment for GDM with metformin or insulin, rather than diet alone^7^.

The objectives of this study were to examine the cardiovascular profile at 5 months postpartum of women who developed GDM, and to determine whether this profile differs between those with dysglycemia and those who are normoglycemic.

## METHODS

### Study design and participants

At the Fetal Medicine Unit of King's College Hospital, London, UK, women attend for routine fetal ultrasound examination at 12, 20 and 36 weeks' gestation. Data on pregnancy outcomes are collected from the hospital maternity records or from the general practitioners who provide primary care for the women.

During a 17‐month period from September 2023 to January 2025, we invited all 956 women presenting to our center who developed GDM to attend a postnatal clinic. In total, 770 (80.5%) women attended the postnatal clinic at a median of 5.0 (interquartile range (IQR), 4.4–7.0) months after delivery and underwent all the investigations outlined below. Women gave written informed consent to participate in the study, which was approved by the National Health Service Research Ethics Committee (reference number: 18/NI/0013; Integrated Research Application System ID: 237936).

At the postnatal clinic visit, we recorded maternal demographic characteristics and obstetric and medical history, including: age; self‐reported ethnicity; family history of DM; parity (parous if any births ≥ 24 weeks' gestation or nulliparous if no such births); GDM in previous pregnancy; gestational age at GDM diagnosis; and treatment required for glycemic control in GDM. Additionally, we measured the weight (in kg) and height (in m) of the women and calculated their body mass index (BMI), and their upper arm and waist circumference (in cm). We performed a 75‐g oral glucose tolerance test (OGTT) and obtained venous blood for measurement of hemoglobin A1c (HbA1c) (in mmol/mol), serum triglycerides (in mmol/L), serum cholesterol (in mmol/L) and serum high‐density lipoprotein (HDL) cholesterol (in mmol/L). OGTT was performed in accordance with the World Health Organization (WHO) guidelines^8^, using a glucose load containing the equivalent of 75 g of anhydrous glucose dissolved in water. Total cholesterol, HDL cholesterol, triglycerides and plasma glucose were all measured using Cobas 8000 instruments (Roche AG, Basel, Switzerland). HbA1C was measured using the International Federation of Clinical Chemistry and Laboratory Medicine assay. All laboratory assays were performed according to standard operating procedures.

### Diagnosis of dysglycemia

The outcome measure was dysglycemia, which was defined as prediabetes or Type‐2 DM. Prediabetes was defined according to the criteria of the American Diabetes Association^9^, as HbA1c between 39 and 47 mmol/mol, fasting plasma glucose between 5.6 and 6.9 mmol/L or impaired glucose tolerance (2‐h blood glucose during OGTT) between 7.8 and 11 mmol/L. Type‐2 DM was defined using the WHO criteria as HbA1c ≥ 48 mmol/mol, fasting plasma glucose ≥ 7.0 mmol/L, 2‐h blood glucose during OGTT of ≥ 11.1 mmol/L or random plasma glucose ≥ 11.1 mmol/L in a patient with symptoms of hyperglycemia^8^. Fasting was defined as no caloric intake for at least 8 h.

### Cardiovascular measurements

#### Ophthalmic arteries

Assessment of the ophthalmic arteries, as a measure of small vessel peripheral circulation, was carried out using a 7.5‐MHz linear transducer (Canon Aplio i900 PLT‐704SBT linear probe; Canon Medical Systems Europe BV, Zoetermeer, The Netherlands). Flow velocity waveforms from the left and right ophthalmic arteries were obtained twice in each eye, with participants in the supine position after approximately 5 min of rest, and the average peak systolic velocity (PSV) ratio of the four measurements was calculated^10^.

#### Aortic stiffness

Aortic stiffness was assessed directly by measuring carotid‐to‐femoral pulse‐wave velocity (PWV). Participants were studied in the supine position after approximately 5 min of rest. Measurements were obtained using the Vicorder device (Skidmore Medical Ltd., Bristol, UK), which measures the time taken for the arterial pulse wave to travel from the carotid to the femoral artery. This device measures simultaneous pressure waveforms by a volume displacement technique, using blood pressure cuffs placed around the neck to pick up the carotid pulse wave and around the right upper thigh to measure the femoral pulse wave in real time over at least 10 heartbeats. Both cuffs are automatically inflated and the corresponding oscillometric signal is analyzed to accurately measure in real time the pulse time delay and consequent PWV. To calculate transit time, the Vicorder software automatically marks the steepest ascending part of the pulse wave (maximum systolic upstroke) and uses a definite timeframe to detect the nadir of the wave. The shift in time between the marked areas on the carotid and femoral pulse waves, which is the transit time, is detected by cross‐correlation. The distance from the carotid and femoral pressure cuffs was measured using a tape. To account for differences in abdominal circumference associated with pregnancy and to reduce variability and error in distance assessment, all measurements were performed from the suprasternal notch to the right shoulder and from there to the midpoint of the blood pressure cuff on the thigh. PWV was expressed in m/s^11^.

#### Pulse‐wave analysis

The brachial artery pulse waveform was also obtained oscillometrically and analyzed using the Vicorder device. Participants were studied in the supine position after approximately 5 min of rest. By applying a brachial‐to‐aortic transfer function, the aortic waveform was generated. Central systolic and diastolic blood pressure, cardiac output and total peripheral resistance (TPR) were derived using the pulse‐wave analysis software. Augmentation pressure was obtained and augmentation index, an indirect measure of arterial stiffness, was expressed as a percentage of central pulse pressure.

#### Echocardiography

Maternal cardiovascular assessment was performed using two‐dimensional and Doppler transthoracic echocardiography. Measurements were taken at rest in the left lateral decubitus position, and data were acquired during unforced expiration. The protocol included standard parasternal and apical views acquired using a Canon Aplio i900 scanner (Canon Medical Systems Europe BV) as per the American Society of Echocardiography and the European Association of Cardiovascular Imaging recommendations^12^, ^13^. Left ventricular systolic function was assessed using M‐mode in the parasternal long‐axis view to calculate fractional shortening and ejection fraction. Ejection fraction was estimated using the Teicholz method. Speckle‐tracking analysis was performed in the four‐chamber, two‐chamber and three‐chamber views to calculate left ventricular global longitudinal systolic function. Increased negative values denote increased deformation and improved myocardial strain.

From structural left ventricular indices, left ventricular mass (LVM) was calculated using the Devereux formula, which uses measurements of the M‐mode applied in the parasternal long axis^14^. Values were indexed to body surface area. Relative wall thickness (RWT) was calculated using the formula: (2 × posterior wall thickness)/left ventricular internal diameter at end‐diastole. LVM was categorized as either concentric (RWT > 0.42) or eccentric (RWT ≤ 0.42) hypertrophy, according to the American Society of Echocardiography and European Association of Cardiovascular Imaging guidelines^14^.

Left ventricular diastolic function was assessed using pulsed‐wave and tissue Doppler. The mitral inflow–velocity pattern was recorded in the apical four‐chamber view, with the pulsed‐wave Doppler sample volume positioned at the tips of the mitral leaflets. The mitral peak early (E) and late (A) diastolic flow velocities were measured, and the E/A ratio was calculated.

Tissue Doppler recordings were obtained at the septal and lateral aspects of the basal left ventricle at the junction with the mitral valve annulus in the apical four‐chamber view. Analysis was performed for the peak systolic annular velocity (S) and the early (e′) and late (a′) mitral annulus diastolic velocities. Using the mitral inflow–velocity curve and the e′ velocity obtained from the septal and lateral aspects of the mitral annulus, the average E/e′ (septal and lateral) ratio was calculated.

The isovolumic contraction (IVCT) and relaxation (IVRT) times were measured from tissue Doppler and left ventricular ejection time (ET) was measured. The myocardial performance index was calculated using the formula (IVCT + IVRT)/ET. The longitudinal function of the right ventricle was assessed by measuring tricuspid annular plane systolic excursion in the four‐chamber view, using M‐mode across the lateral aspect of the tricuspid valve.

Hemodynamic measurements included assessment of cardiac output and TPR. Cardiac output was calculated from stroke volume (derived from the left ventricular outflow tract velocity–time integral) multiplied by the heart rate. TPR was calculated as (mean arterial pressure × 80)/cardiac output.

### Statistical analysis

Quantitative variables were presented as a median (IQR) and qualitative variables were presented as *n* (%). Continuous variables were compared between normoglycemic and dysglycemic women using the Student's *t*‐test or the median test. Differences in the frequencies of categorical variables between those with normoglycemia and those with dysglycemia were tested using the chi‐square test. Quantitative variables were categorized by the quartiles of the whole cohort.

Backward multivariable logistic regression was performed to assess which factors were associated with dysglycemia. Prior to the regression analysis, continuous variables, such as age and BMI, were centered by subtracting the median from each value. In the first step, we included maternal demographic characteristics, variables from the medical history and pregnancy outcome, and findings from the postnatal visit, including cardiovascular indices. In the second step, logistic regression was carried out using biologically relevant variables and those with *P* < 0.1 in the backward elimination step. The relative effect of each variable was calculated as adjusted odds ratio (aOR) with 95% CI. The calibration of the model was evaluated using the Hosmer–Lemeshow test and its discrimination was evaluated using receiver‐operating‐characteristics (ROC) curves.

All statistical tests were two‐sided. *P* < 0.05 was considered statistically significant. The statistical software SPSS version 29 (IBM Corp., Armonk, NY, USA) was used for data analysis.

## RESULTS

The characteristics of the study population are summarized in Table 1. At postnatal follow‐up at a median of 5 months postpartum, dysglycemia was diagnosed in 402 (52.2%) of the 770 women who attended, including 368 (47.8%) with prediabetes and 34 (4.4%) with Type‐2 DM.

**Table 1:** Demographic, pregnancy and postnatal characteristics of study population of 770 women who had developed gestational diabetes mellitus (GDM) and attended postnatal follow‐up

Characteristics	All (*n* = 770)	Normoglycemia (*n* = 368)	Dysglycemia (*n* = 402)	*P* *
*Demographic and pregnancy characteristics*				
Age (years)	35.1 (31.8–38.2)	35.0 (32.0–38.2)	35.2 (31.4–38.1)	0.885
BMI at 11–13 weeks' gestation (kg/m^2^)	27.7 (23.6–32.8)	26.5 (23.3–31.5)	28.5 (24.3–33.6)	0.001
Ethnicity				< 0.001
White	352 (45.7)	211 (57.3)	141 (35.1)	
Black	185 (24.0)	58 (15.8)	127 (31.6)	
South Asian	138 (17.9)	61 (16.6)	77 (19.2)	
East Asian	59 (7.7)	25 (6.8)	34 (8.5)	
Mixed	36 (4.7)	13 (3.5)	23 (5.7)	
Family history of DM†	403 (52.3)	179 (48.6)	224 (55.7)	0.049
Parity				
Nulliparous	344 (44.7)	168 (45.7)	176 (43.8)	0.602
Parous	426 (55.3)	200 (54.3)	226 (56.2)	
With previous GDM	122/426 (28.6)	45/200 (22.5)	77/226 (34.1)	0.009
Without previous GDM	304/426 (71.4)	155/200 (77.5)	149/226 (65.9)	
Gestational age at GDM diagnosis				< 0.001
< 24 weeks	144/762 (18.9)	36/361 (10.0)	108/401 (26.9)	
≥ 24 weeks	618/762 (81.1)	325/361 (90.0)	293/401 (73.1)	
Treatment for GDM				< 0.001
Diet	315 (40.9)	182 (49.5)	133 (33.1)	
Metformin	293 (38.1)	136 (37.0)	157 (39.1)	
Insulin (with or without metformin)	162 (21.0)	50 (13.6)	112 (27.9)	
*Characteristics at postnatal visit*				
Time interval from delivery (months)	5.1 (4.4–6.7)	5.1 (4.4–6.6)	5.2 (4.4–6.7)	0.716
BMI (kg/m^2^)	28.1 (23.8–32.5)	26.8 (23.4–30.9)	29.1 (24.7–34.1)	< 0.001
Waist circumference (cm)	90.0 (81.0–100.0)	88.0 (80.0–97.0)	93.0 (82.3–102.0)	0.003
Upper arm circumference (cm)	29.0 (26.0–33.0)	28.0 (26.0–31.0)	30.0 (27.0–34.0)	< 0.001
OGTT				
Plasma glucose at 0 min (mmol/L)	4.7 (4.3–5.2)	4.6 (4.3–4.9)	4.9 (4.4–5.4)	< 0.001
Plasma glucose at 120 min (mmol/L)	6.0 (5.1–7.2)	5.5 (4.8–6.3)	6.6 (5.5–8.1)	< 0.001
Hemoglobin A1c (mmol/mol)	38.0 (36.0–41.0)	36.0 (34.0–37.0)	41.0 (39.0–43.0)	< 0.001
Serum cholesterol (mmol/L)	5.0 (4.4–5.6)	5.0 (4.4–5.7)	4.9 (4.4–5.6)	0.171
Serum HDL cholesterol (mmol/L)	1.5 (1.3–1.8)	1.5 (1.3–1.9)	1.5 (1.2–1.8)	0.019
Serum triglycerides (mmol/L)	0.9 (0.7–1.3)	0.8 (0.6–1.2)	0.9 (0.7–1.3)	0.098

Data are given as median (interquartile range), *n* (%) or *n*/*N* (%).

* Comparison between groups with normoglycemia and dysglycemia.

† First‐ or second‐degree relative.

DM, diabetes mellitus; HDL, high‐density lipoprotein; OGTT, oral glucose tolerance test.

Women with dysglycemia, compared to the normoglycemic group, had a higher median BMI at 11–13 weeks' gestation (28.5 (IQR, 24.3–33.6) kg/m^2^ *vs* 26.5 (IQR, 23.3–31.5) kg/m^2^) and a higher prevalence of Black ethnicity (31.6% *vs* 15.8%), first‐ or second‐degree family history of DM (55.7% *vs* 48.6%), GDM in a previous pregnancy (34.1% *vs* 22.5%), diagnosis of GDM < 24 weeks' gestation (26.9% *vs* 10.0%) and use of insulin for the treatment of GDM (27.9% *vs* 13.6%). At the postnatal visit, women with dysglycemia had a higher median BMI (29.1 (IQR, 24.7–34.1) kg/m^2^ *vs* 26.8 (IQR, 23.4–30.9) kg/m^2^), median waist circumference (93.0 (IQR, 82.3–102.0) cm *vs* 88.0 (IQR, 80.0–97.0) cm) and median upper arm circumference (30.0 (IQR, 27.0–34.0) cm *vs* 28.0 (IQR, 26.0–31.0) cm), compared with normoglycemic women.

The cardiovascular findings of the study population are reported in Table 2. In women with dysglycemia, compared to normoglycemic women, there was a higher E/e′ ratio, lower LVM index, and higher central systolic and diastolic blood pressure.

**Table 2:** Cardiovascular measurements at postnatal follow‐up in 770 women who had developed gestational diabetes mellitus

Measurement	All (*n* = 770)	Normoglycemia (*n* = 368)	Dysglycemia (*n* = 402)	*P* *
Diastolic indices				
E (cm/s)	78.3 (69.6–90.2)	77.9 (68.8–88.4)	78.5 (70.5–90.9)	0.611
A (cm/s)	43.9 (37.7–52.6)	43.5 (37.2–51.9)	44.1 (38.3–53.1)	0.387
E/A	1.75 (1.49–2.10)	1.74 (1.49–2.14)	1.75 (1.48–2.08)	0.773
IVRT (m/s)	78.0 (67.0–89.0)	78.0 (67.0–89.0)	78.0 (69.0–89.0)	0.936
E/e′	6.89 (5.89–8.03)	6.63 (5.71–7.78)	7.10 (6.07–8.28)	0.004
Left atrial area (cm^2^)	12.0 (10.4–13.9)	12.1 (10.6–14.1)	11.9 (10.3–13.9)	0.196
Left atrial volume (mL)	30.4 (24.9–37.8)	30.6 (25.1–37.9)	30.4 (24.5–37.8)	0.625
Systolic indices				
S (cm/s)	9.7 (8.6–10.8)	9.8 (8.8–10.6)	9.7 (8.5–10.9)	0.433
GLS (%)	−21.8 (−24.1 to −19.6)	−22.0 (−24.2 to −19.5)	−21.7 (−23.9 to −19.6)	0.363
IVCT (m/s)	72.0 (64.0–83.0)	72.0 (64.0–83.0)	72.0 (64.0–83.0)	0.778
MPI	0.52 (0.45–0.60)	0.52 (0.44–0.60)	0.53 (0.46–0.60)	0.373
Ejection fraction (%)	71.0 (65.7–77.0)	71.1 (64.7–76.8)	71.0 (66.5–77.0)	0.836
Structural index				
LVM index	71.8 (62.9–80.8)	72.6 (64.1–81.2)	70.9 (61.8–80.4)	0.021
Vascular indices				
Ophthalmic artery PSV ratio	0.682 (0.622–0.752)	0.684 (0.621–0.751)	0.680 (0.623–0.752)	0.588
Augmentation index (%)	21.0 (15.0–27.0)	20.0 (15.0–26.0)	22.0 (15.0–27.0)	0.079
Mean PWV (m/s)	7.6 (7.0–8.3)	7.5 (7.0–8.3)	7.6 (7.0–8.4)	0.202
Hemodynamic indices				
Heart rate (bpm)	70.0 (63.0–78.0)	69.0 (62.0–76.0)	70.0 (63.0–79.0)	0.232
Central systolic blood pressure (mmHg)	120 (112–128)	118 (111–126)	121 (113–130)	0.004
Central diastolic blood pressure (mmHg)	63 (58–69)	62 (58–67)	64 (58–69)	0.002
CO (L/min) indexed for BMI per kg/m^2^	0.25 (0.21–29)	0.25 (0.21–0.30)	0.24 (0.20–0.29)	0.331
TPR **(**dynes × s/cm^5^ **)**	0.78 (0.67–0.90)	0.80 (0.68–0.91)	0.77 (0.66–0.89)	0.107
Stroke volume (mL)	100.0 (88.0–114.0)	99.0 (88.0–111.8)	100.0 (89.0–117.0)	0.241

Data are given as median (interquartile range).

* Comparison between groups with normoglycemia and dysglycemia.

A, mitral peak late diastolic flow velocity; BMI, body mass index; CO, cardiac output; E, mitral peak early diastolic flow velocity; e′, early mitral annulus diastolic velocity; GLS, global longitudinal strain; IVCT, isovolumic contraction time; IVRT, isovolumic relaxation time; LVM, left ventricular mass; MPI, myocardial performance index; PSV, peak systolic velocity; PWV, pulse‐wave velocity; S, tissue Doppler peak systolic annular velocity; TPR, total peripheral resistance.

In the univariable analysis (Table S1), the demographic/pregnancy characteristics that had a significant association with dysglycemia were Black, South Asian, East Asian and mixed ethnicity, first‐ or second‐degree family history of DM, GDM in a previous pregnancy, diagnosis of GDM < 24 weeks' gestation, and treatment of GDM with metformin or insulin. The postnatal variables that had a significant association with dysglycemia were BMI, waist and upper arm circumference, serum triglycerides, serum HDL cholesterol, E/e′, LVM index, central systolic and diastolic blood pressure, and TPR.

In the multivariable analysis (Table 3), the demographic/pregnancy characteristics that had a significant association with dysglycemia were Black ethnicity (aOR, 2.61 (95% CI, 1.71–3.98)), South Asian ethnicity (aOR, 1.81 (95% CI, 1.18–2.78)), East Asian ethnicity (aOR, 2.46 (95% CI, 1.35–4.48)) and mixed ethnicity (aOR, 2.68 (95% CI, 1.19–6.04)), diagnosis of GDM < 24 weeks' gestation (aOR, 2.15 (95% CI, 1.34–3.45)), and treatment of GDM with insulin (aOR, 1.96 (95% CI, 1.26–3.05)). The postnatal variables that had a significant association with dysglycemia were BMI (aOR, 1.47 (95% CI, 1.05–2.06)), central diastolic blood pressure (aOR, 1.42 (95% CI, 1.01–2.00)), E/e′ (aOR, 1.31 (95% CI, 1.00–1.80)) and TPR (aOR, 0.61 (95% CI, 0.44–0.86)). The calibration of the model was *P* = 0.397 and the discrimination was indicated by an area under the ROC curve of 0.707 (95% CI, 0.670–0.743).

**Table 3:** Multivariable analysis showing variables associated with dysglycemia at 5 months postpartum in women who had developed gestational diabetes mellitus (GDM)

Predictor	aOR (95% CI)	*P*
*Demographic and pregnancy characteristics*		
Ethnicity		< 0.001
White	1.00 (reference)	
Black	2.61 (1.71–3.98)	< 0.001
South Asian	1.81 (1.18–2.78)	0.007
East Asian	2.46 (1.35–4.48)	0.003
Mixed	2.68 (1.19–6.04)	0.017
Family history of DM*	1.37 (1.00–1.88)	0.051
Parity		
Nulliparous	1.00 (reference)	
Parous with previous GDM	0.98 (0.59–1.62)	0.935
Parous without previous GDM	0.70 (0.50–0.98)	0.039
Gestational age at GDM diagnosis		
≥ 24 weeks	1.00 (reference)	
< 24 weeks	2.15 (1.34–3.45)	0.001
Treatment for GDM		0.012
Diet	1.00 (reference)	
Metformin	1.28 (0.91–1.82)	0.162
Insulin (with or without metformin)	1.96 (1.26–3.05)	0.003
*Findings at postnatal visit*		
BMI (in kg/m^2^) – 28	1.47 (1.05–2.06)	0.025
Central diastolic blood pressure (in mmHg) – 63	1.42 (1.01–2.00)	0.042
E/e′ – 7	1.31 (1.00–1.80)	0.049
TPR (in dynes × s/cm^5^) – 0.8	0.61 (0.44–0.86)	0.004

Variables included were age (in years), ethnicity, family history of diabetes mellitus (DM), treatment for GDM, previous GDM, gestational age at GDM diagnosis (in weeks), postnatal body mass index (BMI) (in kg/m^2^), postnatal E/e′, central diastolic blood pressure (in mmHg), total peripheral resistance (TPR) (in dynes × s/cm^5^), left ventricular mass index, augmentation index and stroke volume (in mL).

* First‐ or second‐degree relative; no family history of GDM was used as reference.

aOR, adjusted odds ratio; E, mitral peak early diastolic flow velocity; e′, early mitral annulus diastolic velocity.

## DISCUSSION

### Main findings

This study of an ethnically heterogeneous group of women from an inner‐city population who had developed GDM demonstrated that, at a median of 5 months postpartum, more than half of women had dysglycemia. Multivariable analysis demonstrated that there was a significant association of dysglycemia with Black, South Asian, East Asian and mixed ethnicity, and severe GDM, reflected by GDM diagnosis < 24 weeks' gestation and GDM requiring treatment with insulin, rather than diet alone, for glycemic control. Furthermore, women with persistent dysglycemia at 5 months postpartum have a distinct phenotype which is characterized by increased BMI and signs of cardiovascular strain, including elevated central blood pressure and reduced left ventricular relaxation. These findings suggest that among women who developed GDM, those with persistent dysglycemia are also the ones that appear to be at increased cardiovascular risk.

### Comparison with previous studies and interpretation of results

Increased BMI is a well‐described risk factor for both GDM and Type‐2 DM^15^, ^16^. Our findings show that women with postpartum dysglycemia, compared to those without, not only have an increased BMI but also an increased waist circumference. This is an indicator of abdominal adiposity which is strongly linked to insulin resistance, Type‐2 DM and cardiovascular disease^17^, ^18^, ^19^, ^20^. HbA1c levels were elevated, confirming dysglycemia, while lipid analysis revealed slightly reduced HDL cholesterol and a trend toward elevated fasting triglycerides, suggesting the presence of mild metabolic abnormalities.

These findings align with previous studies^21^, ^22^, ^23^. For instance, the SWIFT cohort study, which included 1035 women diagnosed with GDM, reported that elevated triglycerides and decreased levels of specific sphingolipids were associated with an increased risk of progression to Type‐2 DM^21^. Similarly, in another study of 306 women who had developed GDM and were followed up at 2 months and up to 1 year following delivery, differences in HDL cholesterol and triacylglycerol concentrations were seen in those with dysglycemia^23^.

Several studies have examined the impact of GDM on maternal cardiac function during the postpartum period and later in life^24^, ^25^. In our previous longitudinal study of 73 women with GDM and 73 women with an uncomplicated pregnancy, our group reported that women with GDM exhibited subtle impairments in both systolic and diastolic left ventricular function during late pregnancy, which persisted 6 months after delivery, albeit to a lesser degree^26^. In the current study, we observed that women who had developed GDM and dysglycemia at approximately 5 months postpartum, compared to those without dysglycemia, had reduced left ventricular relaxation, indicated by an elevated tissue Doppler E/e′ ratio. An increased E/e′ ratio is a well‐established marker of elevated left ventricular filling pressure and is associated with long‐term cardiovascular risk, including heart failure and arrhythmia, even when systolic function is preserved^27^, ^28^, ^29^. This finding suggests that women with dysglycemia, compared to those without, also have a more aggravated cardiovascular profile. However, no causal relationships can be established from the design of our study.

It is unclear whether the noted cardiac remodeling is due to inherent myocardial abnormalities in these women or whether it represents an inadequate adaptive response to increased blood pressure, abnormal metabolic profile or vascular disease^30^. In our study, women with dysglycemia exhibited increased central systolic and diastolic blood pressure, while other hemodynamic and vascular indices remained preserved. PWV, a marker of aortic stiffness, did not differ between the two groups. Additionally, markers of peripheral arterial disease, such as the ophthalmic artery PSV ratio, were similar in those with and those without dysglycemia.

Typically, increased central systolic blood pressure would trigger left ventricular hypertrophy as a compensatory mechanism^31^; the absence, or even reversal, of this response, as noted in women with dysglycemia in our study, suggests an abnormal adaptation. This pattern indicates an environment of high afterload and diastolic dysfunction without the expected compensatory myocardial hypertrophy. However, in our multivariable analysis, the only cardiovascular associations with dysglycemia were increased central diastolic blood pressure, increased left ventricular diastolic pressure and reduced TPR. This mismatch might indicate that while the central vasculature (i.e. closer to the heart) is experiencing higher pressures, the peripheral vessels are dilating, possibly as a compensatory mechanism, but this is not sufficient since the diastolic filling pressures in the heart are increasing. A recent meta‐analysis of 15 cohort studies, including nearly 4 million individuals (175 378 with GDM and 3 784 142 without GDM), reported 106 560 cases of systemic hypertension over follow‐up periods ranging from 2 to 20 years after delivery, linking GDM with long‐term hypertension risk^32^; this further supports our findings which link increased central blood pressure to dysglycemia and later cardiovascular disease.

### Strengths and limitations

Our study benefits from a large sample size drawn from an ethnically diverse population, with a high representation of Black and South Asian women; these ethnic groups are known to be at greater risk for Type‐2 DM and cardiometabolic disorders. This enhances the generalizability of our findings to these high‐risk groups.

Several limitations should be considered when interpreting our results. First, comparisons were made based solely on dysglycemia status and we did not include women with an uncomplicated pregnancy. Consequently, while the dysglycemia group is at higher risk for Type‐2 DM or cardiovascular disease, this does not imply that the normoglycemic group is free from residual risk. Additionally, given the ethnic composition of our sample, the findings may not be fully generalizable to predominantly White populations. We used diabetic treatment type as a proxy for GDM severity, but it is possible that some patients might have been misclassified. However, such bias would have diminished, rather than enhanced, the noted associations with dysglycemia. Finally, the possibility of residual confounding due to unmeasured variables cannot be excluded.

Despite these limitations, our results suggest that abnormalities in the metabolic and cardiac phenotypes track together. These findings underscore the need for tailored postpartum interventions that address modifiable risk factors, such as dietary modifications aligned with culturally specific food preferences, and physical activity programs that are both acceptable and feasible for high‐risk populations.

### Conclusion

Women who developed GDM and had dysglycemia at 5 months postpartum have increased central blood pressure and evidence of adverse cardiovascular remodeling with increased left ventricular filling pressure. These findings suggest that women with dysglycemia also have a worse cardiovascular profile and need to be closely monitoring to avoid early development of heart failure. Further studies are needed to assess whether targeting weight loss with lifestyle or pharmacological interventions can reduce the risk not only for Type‐2 DM, but also for later cardiovascular disease.

## Supporting information


**Table S1** Univariable analysis of variables associated with dysglycemia at 5 months postpartum in women who had developed gestational diabetes mellitus

## Data Availability

Research data are not shared.

## References

[1] Vounzoulaki E , Khunti K , Abner SC , Tan BK , Davies MJ , Gillies CL . Progression to type 2 diabetes in women with a known history of gestational diabetes: systematic review and meta‐analysis. BMJ. 2020;369:m1361. 32404325 10.1136/bmj.m1361 PMC7218708

[2] Kim C , Newton KM , Knopp RH . Gestational diabetes and the incidence of type 2 diabetes: a systematic review. Diabetes Care. 2002;25:1862‐1868. 12351492 10.2337/diacare.25.10.1862

[3] Kitzmiller JL , Dang‐Kilduff L , Taslimi MM . Gestational diabetes after delivery. Short‐term management and long‐term risks. Diabetes Care. 2007;30:S225‐S235. 17596477 10.2337/dc07-s221

[4] Kramer CK , Campbell S , Retnakaran R . Gestational diabetes and the risk of cardiovascular disease in women: a systematic review and meta‐analysis. Diabetologia. 2019;62:905‐914. 30843102 10.1007/s00125-019-4840-2

[5] Tobias DK , Stuart JJ , Li S , et al. Association of history of gestational diabetes with long‐term cardiovascular disease risk in a large prospective cohort of US women. JAMA Internal Med. 2017;177:1735‐1742. 29049820 10.1001/jamainternmed.2017.2790 PMC5820722

[6] Yu Y , Soohoo M , Sørensen HT , Li J , Arah OA . Gestational diabetes mellitus and the risks of overall and type‐specific cardiovascular diseases: a population‐and sibling‐matched cohort study. Diabetes Care. 2022;45:151‐159. 34764208 10.2337/dc21-1018 PMC8753767

[7] Gómez Fernández C , Golubic R , Mitsigiorgi R , Mansukhani T , Car J , Nicolaides KH . Predictors of cardiometabolic health a few months postpartum in women who had developed gestational diabetes. Nutrients. 2025;17:390. 39940248 10.3390/nu17030390 PMC11820877

[8] Organization WHO . Diagnosis and management of type 2 diabetes (HEARTS‐D). Geneva. 2020 Available at: https://www.who.int/publications/i/item/who‐ucn‐ncd‐20.1. Accessed 2024

[9] Committee ADAPP, Committee: ADAPP . 2. Classification and diagnosis of diabetes: standards of medical care in diabetes—2022. Diabetes Care. 2022;45:S17‐S38. 34964875 10.2337/dc22-S002

[10] Sarno M , Wright A , Vieira N , Sapantzoglou I , Charakida M , Nicolaides K . Ophthalmic artery Doppler in combination with other biomarkers in prediction of pre‐eclampsia at 35–37 weeks' gestation. Ultrasound Obstet Gynecol. 2021;57:600‐606. 33073902 10.1002/uog.23517

[11] Mansukhani T , Arechvo A , Cecchini F , et al. Vascular phenotype at 35‐37 weeks' gestation in women with gestational diabetes mellitus. Ultrasound Obstet Gynecol. 2023;61:386‐391. 36173400 10.1002/uog.26077

[12] Othman F , Abushahba G , Salustri A . Adherence to the American society of echocardiography and European association of cardiovascular imaging recommendations for the evaluation of left ventricular diastolic function by echocardiography: a quality improvement project. J Am Soc Echocardiogr. 2019;32:1619‐1621. 31668767 10.1016/j.echo.2019.09.005

[13] Lang RM , Badano LP , Mor‐Avi V , et al. Recommendations for cardiac chamber quantification by echocardiography in adults: an update from the American Society of Echocardiography and the European Association of Cardiovascular Imaging. Eur Heart J Cardiovasc Imaging. 2015;16:233‐270. 25712077 10.1093/ehjci/jev014

[14] Lee L , Cotella JI , Miyoshi T , et al. Normal values of left ventricular mass by 2D and 3D echocardiography: results from the world alliance societies of echocardiography normal values study. J Am Soc Echocardiogr. 2023;36:533‐542. 36584904 10.1016/j.echo.2022.12.016

[15] Baptiste‐Roberts K , Barone BB , Gary TL , et al. Risk factors for type 2 diabetes among women with gestational diabetes: a systematic review. Am J Med. 2009;122(207–214):e204. 10.1016/j.amjmed.2008.09.034 PMC6393925 19272478

[16] Yashi K , Daley SF . Obesity and Type 2 Diabetes. StatPearls. StatPearls Publishing; 2024. 37276290

[17] Ritchie S , Connell J . The link between abdominal obesity, metabolic syndrome and cardiovascular disease. Nutr Metab Cardiovasc Dis. 2007;17:319‐326. 17110092 10.1016/j.numecd.2006.07.005

[18] Ramírez‐Manent JI , Jover AM , Martinez CS , Tomás‐Gil P , Martí‐Lliteras P , López‐González ÁA . Waist circumference is an essential factor in predicting insulin resistance and early detection of metabolic syndrome in adults. Nutrients. 2023;15:257. 36678129 10.3390/nu15020257 PMC9861022

[19] Jayedi A , Soltani S , Motlagh SZ‐T , et al. Anthropometric and adiposity indicators and risk of type 2 diabetes: systematic review and dose‐response meta‐analysis of cohort studies. BMJ. 2022;376:e067516. 35042741 10.1136/bmj-2021-067516 PMC8764578

[20] Ke J‐F , Wang J‐W , Lu J‐X , Zhang Z‐H , Liu Y , Li L‐X . Waist‐to‐height ratio has a stronger association with cardiovascular risks than waist circumference, waist‐hip ratio and body mass index in type 2 diabetes. Diabetes Res Clin Pract. 2022;183:109151. 34863718 10.1016/j.diabres.2021.109151

[21] Khan SR , Rost H , Cox B , et al. Heterogeneity in early postpartum metabolic profiles among women with GDM who progressed to type 2 diabetes during 10‐year follow‐up: the SWIFT study. medRxiv. 2023;2023.06.13.23291346.

[22] Wang G , Buckley JP , Bartell TR , Hong X , Pearson C , Wang X . Gestational diabetes mellitus, postpartum lipidomic signatures, and subsequent risk of type 2 diabetes: a lipidome‐wide association study. Diabetes Care. 2023;46:1223‐1230. 37043831 10.2337/dc22-1841 PMC10234741

[23] Prados M , Flores‐Le Roux JA , Benaiges D , et al. Previous gestational diabetes increases atherogenic dyslipidemia in subsequent pregnancy and postpartum. Lipids. 2018;53:387‐392. 29732563 10.1002/lipd.12040

[24] Thirunavukarasu S , Ansari F , Kotha S , et al. Cardiac structural, functional and energetic assessments during and after pregnancy in women with gestational diabetes mellitus, preeclampsia and healthy pregnancy. Am J Obstet Gynecol. 2024;S0002‐9378(24):01157–8. 10.1016/j.ajog.2024.11.018 39581289

[25] Minhas AS , Countouris M , Ndumele CE , et al. Association of gestational diabetes with subclinical cardiovascular disease. JACC Adv. 2024;3:101111. 39105123 10.1016/j.jacadv.2024.101111 PMC11299583

[26] Aguilera J , Sanchez Sierra A , Abdel Azim S , Georgiopoulos G , Nicolaides KH , Charakida M . Maternal cardiac function in gestational diabetes mellitus at 35–36 weeks' gestation and 6 months postpartum. Ultrasound Obstet Gynecol. 2020;56:247‐254. 32530101 10.1002/uog.22118

[27] Ratneswaren A , Wu T , Kaura A , et al. Tissue Doppler echocardiography predicts long‐term cardiovascular mortality: the Anglo‐Scandinavian Cardiac Outcomes Trial (ASCOT) legacy 20‐year follow‐up study. Open Heart. 2025;12:e002795. 39904554 10.1136/openhrt-2024-002795 PMC11795408

[28] Harada T , Obokata M , Kagami K , et al. Utility of E/e′ ratio during low‐level exercise to diagnose heart failure with preserved ejection fraction. JACC Cardiovasc Imaging. 2023;16:145‐155. 36752422 10.1016/j.jcmg.2022.10.024

[29] Wu VC‐C , Huang Y‐C , Wang C‐L , et al. Association of echocardiographic parameter E/e′with cardiovascular events in a diverse population of inpatients and outpatients with and without cardiac diseases and risk factors. J Am Soc Echocardiogr. 2023;36:284‐294. 36332804 10.1016/j.echo.2022.10.016

[30] Lv J , Liu Y , Yan Y , et al. Relationship between left ventricular hypertrophy and diabetes is likely bidirectional: a temporality analysis. J Am Heart Assoc. 2023;12:e028219. 36892057 10.1161/JAHA.122.028219 PMC10111543

[31] Weber T , Wassertheurer S , Schmidt‐Trucksäss A , et al. Relationship between 24‐hour ambulatory central systolic blood pressure and left ventricular mass: a prospective multicenter study. Hypertension. 2017;70:1157‐1164. 29061725 10.1161/HYPERTENSIONAHA.117.09917

[32] Liu X , Nianogo RA , Janzen C , et al. Association between gestational diabetes mellitus and hypertension: a systematic review and meta‐analysis of cohort studies with a quantitative bias analysis of uncontrolled confounding. Hypertension. 2024;81:1257‐1268. 38501243 10.1161/HYPERTENSIONAHA.123.22418

