# Supplementary material for: Cardiovascular function in women with previous gestational diabetes mellitus and dysglycemia at 5 months postpartum
Source: Ultrasound Obstet Gynecol. 2025 Jun 30;66(2):155–62. doi: 10.1002/uog.29285 (PMC12317300; doi:10.1002/uog.29285)
Supplement: Supplementary file 1 — Table S1 Univariable analysis of variables associated with dysglycemia at 5 months postpartum in women who had developed gestational diabetes mellitus [file UOG-66-155-s001.docx]

**Table S1** Univariable analysis reporting associations with dysglycemia at 5 months postpartum in women who had gestational diabetes mellitus (GDM)

| **Predictor** | **OR (95% CI)** | **p-value** |
| --- | --- | --- |
| **Demographic and pregnancy characteristics** |  |  |
| Age - 35 (years) | 1.00 (0.75-1.33) | 0.996 |
| Ethnicity |  | <0.001 |
| White (reference) | 1.00 |  |
| Black | 3.28 (2.25-4.78) | <0.001 |
| South Asian | 1.89 (1.27-2.81) | 0.002 |
| East Asian | 2.04 (1.16-3.56) | 0.013 |
| More than one | 2.65 (1.30-5.40) | 0.007 |
| 1^st^ or 2^nd^ degree family history of diabetes  (reference: no family history) | 1.33 (1.00-1.77) | 0.049 |
| Parity |  | 0.030 |
| Nulliparous (reference) | 1.00 |  |
| Parous with previous GDM | 1.63 (1.07-2.50) | 0.023 |
| Parous without previous GDM | 0.92 (0.68-1.26) | 0.614 |
| Gestational age at diagnosis of GDM |  |  |
| ≥ 24 weeks (reference) | 1.00 |  |
| <24 weeks | 3.33 (2.21-5.01) | <0.001 |
| Treatment for GDM |  | <0.001 |
| Diet (reference) | 1.00 |  |
| Metformin | 1.58 (1.15-2.18) | 0.005 |
| Insulin (+/- metformin) | 3.07 (2.05-4.58) | <0.001 |
| **Postnatal visit** |  |  |
| Postnatal BMI – 28 (kg/m^2^) | 1.94 (1.46-2.59) | <0.001 |
| Upper arm circumference – 29 (cm) | 1.85 (1.39-2.46) | <0.001 |
| Waist circumference – 90 (cm) | 1.69 (1.27-2.25) | <0.001 |
| Serum Cholesterol – 5.0 (mmol/L) | 0.86 (0.65-1.14) | 0.289 |
| Serum triglycerides – 0.9 (mmol/L) | 1.41 (1.06-1.87) | 0.020 |
| Serum HDL-Cholesterol – 1.5 (mmol/L) | 0.68 (0.51-0.90) | 0.008 |
| **Cardiac measurements** |  |  |
| Mitral valve E/A – 1.75 | 1.01 (0.76-1.34) | 0.963 |
| Isovolumic contraction time - 72 (msec) | 0.84 (0.63-1.12) | 0.231 |
| Isovolumic relaxation time IVRT – 78 (msec) | 1.05 (0.79-1.39) | 0.740 |
| Myocardial performance index – 0.5 | 1.19 (0.89-1.58) | 0.247 |
| Mitral valve E/e' – 7.0 | 1.61 (1.21-2.14) | 0.001 |
| Mitral valve s mean – 9.7 | 0.93 (0.70-1.23) | 0.598 |
| Left atrial area – 12 (cm2) | 0.84 (0.63-1.11) | 0.224 |
| Left atrial volume – 30 (mL) | 0.98 (0.74-1.30) | 0.899 |
| Left ventricular mass index – 72 | 0.74 (0.55-0.97) | 0.032 |
| Ejection fraction Teicholz - 71 (%) | 0.98 (0.72-1.30) | 0.880 |
| Global longitudinal strain – (-21.8) (%) | 1.13 (0.85-1.50) | 0.412 |
| Ophthalmic artery PSV2/PSV1 – 0.7 | 1.07 (0.81-1.43) | 0.631 |
| Heart rate – 70 (bpm) | 1.22 (0.91-1.62) | 0.182 |
| Central systolic blood pressure – 120 (mmHg) | 1.64 (1.23-2.19) | <0.001 |
| Central diastolic blood pressure – 63 (mmHg) | 1.53 (1.14-2.03) | 0.004 |
| Augmentation index – 21 (%) | 1.29 (0.97-1.72) | 0.083 |
| Cardiac output (L/min) index for BMI - 0.250 (L/min per Kg/m^2^) | 1.18 (0.78-1.79) | 0.436 |
| Total peripheral resistance – 0.8 **(**dynes ×sec/cm^5^**)** | 0.74 (0.55-0.98) | 0.037 |
| Stroke volume – 100 (mL) | 1.27 (0.95-1.69) | 0.103 |
| Mean pulse wave velocity – 7.6 (m/sec) | 1.20 (0.90-1.60) | 0.216 |
